# The corticospinal tract structure of collagen/silk fibroin scaffold implants using 3D printing promotes functional recovery after complete spinal cord transection in rats

**DOI:** 10.1007/s10856-021-06500-2

**Published:** 2021-03-22

**Authors:** Xiao-Hong Li, Xiang Zhu, Xiao-Yin Liu, Hai-Huan Xu, Wei Jiang, Jing-Jing Wang, Feng Chen, Sai Zhang, Rui-Xin Li, Xu-Yi Chen, Yue Tu

**Affiliations:** 1 Academy of Medical Engineering and Translational Medicine, Tianjin University, Tianjin, 300072 China; 2 Tianjin Key Laboratory of Neurotrauma Repair, Pingjin Hospital Brain Center, Characteristic Medical Center of PAPF, Tianjin, 300162 China; 3 Henan provincial people’s hospital of southeast branch, Zhu ma dian, 463500 China; 4 Tianjin Medical University, Tianjin, 300070 China; 5 Emergency Medical Center, Beijing Chaoyang Integrative medicine, Beijing, 100191 China; 6 Central Laboratory, Tianjin Stomatological Hospital, Tianjin, 300041 China

## Abstract

No effective treatment has been established for nerve dysfunction caused by spinal cord injury (SCI). Orderly axonal growth at the site of spinal cord transection and creation of an appropriate biological microenvironment are important for functional recovery. To axially guiding axonal growth, designing a collagen/silk fibroin scaffold fabricated with 3D printing technology (3D-C/SF) emulated the corticospinal tract. The normal collagen/silk fibroin scaffold with freeze-drying technology (C/SF) or 3D-C/SF scaffold were implanted into rats with completely transected SCI to evaluate its effect on nerve repair during an 8-week observation period. Electrophysiological analysis and locomotor performance showed that the 3D-C/SF implants contributed to significant improvements in the neurogolical function of rats compared to C/SF group. By magnetic resonance imaging, 3D-C/SF implants promoted a striking degree of axonal regeneration and connection between the proximal and distal SCI sites. Compared with C/SF group, rats with 3D-C/SF scaffold exhibited fewer lesions and disordered structures in histological analysis and more GAP43-positive profiles at the lesion site. The above results indicated that the corticospinal tract structure of 3D printing collagen/silk fibroin scaffold improved axonal regeneration and promoted orderly connections within the neural network, which could provided a promising and innovative approach for tissue repair after SCI.

## Introduction

Spinal cord injury (SCI) is a major public health problem and a devastating condition that leads to high mortality rates and loss of respiratory, autonomic, and sensory-motor functions [1]. Current interventions include the administration of anti-inflammatory agents, surgical decompression, and stabilization of the vertebral column, all of which are targeted at preventing further neuronal death and functional deficits after SCI [2]. However, no effective therapy that substantially promotes tissue regeneration and restoration of functional deficits has been established. Thus, nerve repair after SCI remains a challenging clinical problem. Spontaneous axonal regeneration is limited by several inhibitory factors after primary and secondary SCI [3, 4]. A major impediment to regeneration is the formation of cysts and glial scarring, which create a gap or physical barrier that restricts axonal regrowth [1, 5]. One potential approach is to rebuild the tissue destroyed by cysts and glial scarring with functional tissue, providing a supportive substrate to guide neurons and break the physical and chemical barrier caused by the loss of spinal cord tissue and glial scarring. Implantation of biomaterial scaffolds may be an optimal alternative for replacement of the lesion cavity and barrier.

The emerging field of tissue engineering has promising therapeutic potential. Biodegradable polymer grafts may have significant therapeutic potential in the surgical repair of SCI [6–8]. Recent therapeutic attempts in the field of regeneration medicine after SCI have therefore focused on the use of engineering materials that mimic the spinal cord niche. Biologic scaffold materials are used to repair and reconstruct injured or missing tissues. A variety of scaffold materials, either naturally derived or synthetic, have been tested [9–11]. Despite some promising results, the development of tissue-engineered scaffolds suitable for SCI is still at an early stage [8, 12]. Questions remain regarding the optimal structure and composition of the scaffold. The fabrication technique, controlled microarchitecture, and biomaterials composition are key factors for effective polymer implants and suitable scaffolds for SCI.

Implant engineering has been limited by fabrication technology. The diameter of spinal cord implants, particularly in animal experiments, is only about 3 mm [7]. Traditional fabrication technology, such as particulate leaching techniques, heat compression, extrusion techniques, and others, are not geared toward the engineering of spinal cord implants with precise internal microarchitecture on a small scale. The recent emergence of three-dimensional (3D) printing, a type of solid free-form fabrication in tissue engineering, provides a novel and feasible method with which to improve implant properties [7, 13]. In 3D printing, a print head creates a 3D polymer substrate comprising a series of two-dimensional (2D) layers. The scaffold parameters can be optimized on a computer workstation [14, 15]. This technique may enable the design and fabrication of implants with novel and precisely defined micro- and macroarchitecture. Therefore, compared with conventional technologies, 3D printing has obvious advantages in the engineering of spinal cord scaffolds.

The engineering of scaffolds of various internal microarchitectures may significantly improve axonal regeneration and ultimately facilitate neurological repair after SCI. The most common implant design in spinal cord transection models is a cylindrical structure with a regular array of cylindrical channels. The hollow internal cylinders within the polymer implant act as guidance channels for axonal regrowth [8]. However, a design with a more creative and bionic internal microarchitecture could be advantageous. The breakage of axon tracts in white matter is the most critical and direct reason for the loss of sensory-motor function after SCI. Thus, the design of a scaffold implant that emulates the white matter and axially oriented for axonal guidance might enhance the specificity of target acquisition and allow for the application of tract-specific growth-enhancing strategies.

In addition to fabrication technology and microarchitecture design, the choice of biomaterials is also important. The ideal materials for spinal cord scaffolds should be biocompatible, exhibit nontoxic degradation, and have suitable mechanical properties. Various biomaterials may be candidates for use in potential therapies after SCI. Collagen, the most important extracellular matrix component in the body, has been widely used for a variety of tissue engineering applications because of its abundance and minimal host immune response [16]. However, the disadvantages of collagen scaffolds are poor mechanical strength and rapid degradation [17, 18]. Silk fibroin (SF), a unique natural protein, is currently utilized as a biomaterial for soft tissue engineering and reconstruction [19, 20]. Incorporation of SF, which is characterized by high mechanical strength, remarkable elasticity, and environmental stability, could compensate for the disadvantages of using a collagen scaffold alone [21–24].

A suitable scaffold with optimal biocompatibility, strength, and precise microarchitecture has not yet been developed. In this study, we aimed to design a functionalized spinal cord scaffold using a conceptually new approach that simulates the framework of white matter. We adopted 3D printing technology with synergistic incorporation of collagen and SF to produce a scaffold with a special architecture that targets important white matter tracts. We hypothesized that this precisely designed scaffold significantly facilitates native axonal regeneration and neurological improvement after SCI.

## Materials and methods

### 3D printing scaffolds

SF was prepared using cocoons according to an established method [25, 26]. The fibroin was placed in 0.5% Na_2_CO_3_ solution at 98–100 °C for 30 min, and this was repeated three times. The fibroin was then dried with a ternary solvent comprising CaCl_2_, CH3CH2OH, and H_2_O in a substance concentration ratio of 1:2:8 at 70 ± 2 °C and stirred to dissolve, After dialysis, filtration, and concentration, a specific concentration of fibroin solution was obtained. Collagen (2.0 mg/ml) was produced based on previous studies [26–28]. The outer membrane of a fresh bovine tendon was cleaned, the fat tissue was removed, and the tendon was crushed and soaked in 0.05 M Tris buffer for 24 h to remove soluble impurities. After centrifugation, the precipitate was added to an acetic acid solution containing pepsin. After full swelling, the supernatant was collected. NaCl solution was then added for salting. The salting precipitate was collected after centrifugation and dialyzed in deionized water for 5 days to obtain a purified collagen gel. The fibroin and collagen were mixed (mass ratio, 1:1). Part of the obtained composite materials were freeze-dried for 24–48 h to obtain the collagen/silk fibroin (C/SF) scaffold, and the other part of composite materials were prepared by 3D printers at −20 C to prepare the 3D printing collagen/silk fibroin (3D-C/SF) scaffold.

The spinal cord model used in this study was designed by SolidWorks software (Dassault Systèmes, Vélizy, France). The pre-designed STL file was imported into the upper computer software to convert STL 3D model into G code. Inner diameter tips of 210 μm were used as a printing nozzle. The printing speed, extrusion speed, and layer thickness were set to be 9 mm/s, 2 mm/min, and 0.1 mm respectively. The compound material of collagen and SF was loaded into the printing cartridge and extruded by printing nozzle, which was cooled rapidly into solid shape. After printing, they were vacuum freeze-dried for 24–48 h to prepare 3D-C/SF scaffold.

### Ultrastructural observations

For scanning electron microscopic observation, the 3D-C/SF scaffolds were washed three times with phosphate-buffered saline (PBS), fixed in 3% glutaraldehyde for 90 min, dehydrated with a graded ethanol series, and freeze-dried for 2 days. The dried samples were coated with gold and examined under a scanning electron microscope (XL30 FEG; Philips, Amsterdam, The Netherlands).

### Mechanical properties

Each set of the 3D-C/SF scaffolds (*n* = 3) was immersed in a 0.01 mol/L PBS solution (pH 7.4) at 37 °C for 24 h until equilibrium. Compressive modulus and strength was tested using an Instron 5865 (Instron, Norwood, MA, USA). The sinusoidal waveform was 5 Hz, preload was 0.1 N, and maximum compressive strain was 10%. The samples were looped three times. A stress-strain curve was drawn to determine the compressive modulus and strength of scaffold.

### X-ray diffractometry

The 3D-C/SF scaffolds were placed in an X-ray diffractometer (D8 Advance; Bruker AXS GmbH, Karlsruhe, Germany) for crystallization analysis. The tube voltage was 40 kV, tube current was 30 mA, target material was CuKα (=0.15406 nm), scanning speed was 5°/min, step width was 0.02°, and diffraction angle was 5–90°.

### Fourier transform infrared spectroscopy

For Fourier transform infrared (FTIR) spectroscopy, the 3D-C/SF scaffold was grinded into a powder using a KBr tablet in an infrared spectrometer (Nicolet 870; Thermo Fisher Scientific, Waltham, MA, USA). The scanning range was 400–4000 cm^−1^.

### Differential scanning calorimetry

The phase transition temperatures of 3D-C/SF scaffolds during heating were analyzed by differential scanning calorimetry (DSC) (DSC822e differential scanning calorimeter; Mettler Toledo, Greifensee, Switzerland). The scaffolds were warmed at a heating rate of 10 K/min under an argon atmosphere from 30 to 400 °C.

### In vivo degradation of scaffold

Adult female SD rats (*n* = 6) were anesthetized by intraperitoneal injection of 5% chloral hydrate at 0.6 ml/100 g. Longitudinal incision of 1 cm in the back near the center of the T10 was made, and 3D-C/SF scaffold was implanted into the cystic space. After 1, 2, 3, and 4 weeks, the scaffold along with the surrounding tissue was removed and scaffold degradation rate was calculated according to the following formula: degradation rate = (W0 − WT)/W0 × 100%. W0 is the quality of materials before degrading. WT is the quality of the residual materials after degrading.

### Spinal cord transection and transplantation

Adult female Sprague-Dawley (SD) rats (250–280 g, *n* = 100) were supplied by the Military Medical Academy Animal Breeding Center. The SD rats were anesthetized by intraperitoneal injection of 5% chloral hydrate at 0.6 ml/100 g. Following laminectomy at the T9 vertebral level, the dorsal surface of the dura mater at the T10 level was exposed by laminectomy [29] and a 3-mm cord segment including visible spinal roots was completely removed at the T10 spinal cord level [30] (Gelderd and Chopin). Immediately after injury, the scaffolds were implanted to fill the lesion gap. All rats were divided into four groups according to different treatments: the sham group (the lamina is open to expose the spinal cord without SCI, *n* = 20), the SCI group (SCI rats without a transplanted scaffold, *n* = 20), the SCI + C/SF group (SCI rats implanted with a C/SF scaffolds by freeze-drying technology, *n* = 20), and the SCI + 3D-C/SF group (SCI rats implanted with a C/SF scaffolds by 3D printing technology, *n* = 20). The bladders of injured rats were massaged twice daily after surgery until the bladder function was recovered. All experimental protocols and animal handling procedures were approved by the Affiliated Hospital of Logistics University of the Chinese People’s Armed Police Forces and were consistent with the National Institutes of Health Guide for the Care and Use of Laboratory Animals.

### Assessment of locomotor performance

The hind limb motor function in each rat was evaluated once weekly using the Basso, Beattie, and Bresnahan (BBB) open-field locomotor test [31]. This test quantitatively evaluated voluntary movement and body weight support. Individual animals were allowed to move freely for 5 min, and hind limb movements were observed by three examiners. The average score was used for analysis.

### Electrophysiological analysis

Neurophysiology monitoring setup was used to evaluate somatosensory evoked potentials (SEP) and motor evoked potentials (MEP) with evoked potential equipment (Nicolet VikingQuest; Natus Medical Inc., Pleasanton, CA, USA). Two baseline recordings were taken prior to SCI, and one recording per week was taken after SCI. The animals were anesthetized by intraperitoneal injection of 5% chloral hydrate. For SEP, The stimulating electrodes were placed in the upper right quadrant of the intersection of the coronal suture and sagittal suture with the following settings: stimulus intensity, 46 V; stimulation pulse width, 0.2 ms; stimulation frequency, 1 Hz; left leg incubation period, 5.8 ms; volatility, 540.4 μV; right leg incubation period, 6.1 ms; and volatility, 893.5 μV. The recording electrode was placed in the posterior tibial nerve, and the reference electrode was placed in the corresponding part of the skin. Changes in the MEP latency and amplitude were observed. SEP and MEP were recorded to assess the functional status of motor and sensory axonal conduction. The placement positions of the stimulating and recording electrodes were interchangeable during acquisition of MEP.

### Magnetic resonance imaging

Rats were anesthetized with an intraperitoneal injection of 5% chloral hydrate (0.6 mL/100 g). Rats were placed in a dedicated coil with the spinal cord located in the center. The scan was performed in the following order: sagittal T2 spectral fat suppression, sagittal T1 fast spin echo, cross-section T2 fast spin echo, 3D T1 images, and diffusion tensor imaging (DTI). T2 sequence: TR, 4000 ms; TE, 74.0 ms; scan time, 4 m 21 s; slice thickness, 1.5 mm; layer spacing, 0.15 mm; vision (field of view [FOV]), 100 × 100; matrix, 224 × 320 s. T1 sequence: TR, 600 ms; TE, 13.0 ms; scan time, 1 m 32 s; slice thickness, 1.5 mm; layer spacing, 0.15 mm; FOV, 50 × 100; matrix, 112 × 320 s. DTI sequence: TR, 4800 ms; TE, 120 ms; scan time, 3 m 38 s; slice thickness, 3 mm; interlayer spacing, 0.12 mm; FOV, 10^7^ × 10^7^; matrix, 128 × 128 s; *b*-value, 1000 s/mm^2^.

### BDA anterograde tracing of the corticospinal tract

At 6 weeks post injury, the rats underwent anterograde tracing of the corticospinal tract with biotinylated dextran amine (BDA) (*n* = 4). After the rats were anesthetized, they were positioned in a stereotaxic frame. BDA (10%, 10,000 MW; Invitrogen, Eugene, OR, USA) was injected unilaterally into the left and right sensorimotor cortexes at 12 sites (0.5 μL/site) using the following coordinates (in reference to Bregma): (1) 0.5 mm anterior and 2.5 mm lateral, (2) 0.5 mm anterior and 3.5 mm lateral, (3) 1.5 mm anterior and 2.5 mm lateral, (4) 1.5 mm anterior and 3.5 mm lateral, (5) 1.0 mm posterior and 2.5 mm lateral, and (6) 1.0 mm posterior and 3.5 mm lateral. Injections were made 1.5 mm from the surface of the cortex.

### Immunofluorescence staining

After 8 weeks, the rats were transcardially perfused with 100 mL of cold PBS followed by 300 mL of 4% paraformaldehyde in phosphate buffer. The spinal cords were obtained, postfixed overnight in 4% paraformaldehyde, and cryoprotected in 0.1 M phosphate buffer containing 20% and 30% sucrose at 4 °C. Sagittal sections were cut on a freezing microtome set. Primary antibodies (anti-NF (1:200, Abcam, Cambridge, UK) anti-MBP (1:500, Abcam, Cambridge, UK) anti-GAP43 (1:100, Abcam, Cambridge, UK)) was used. The corresponding secondary antibody was obtained from Invitrogen (Carlsbad, CA, USA). The sections were incubated with primary antibodies mixed in 0.3% Triton X-100 overnight at 4 °C, followed by incubation with secondary antibodies. The slides were then examined under a fluorescence microscope.

### Statistical analysis

All statistical analyses were performed using the statistical software SPSS v.19.0 (IBM Corp., Armonk, NY, USA). Data are reported as mean ± standard deviation. When two sets of data were compared, an independent-samples *t* test or two-way analysis of variance was performed. A statistically significant difference was accepted at *P* < 0.05.

## Results

### Design and manufacture of scaffold

**Fig. 1:**
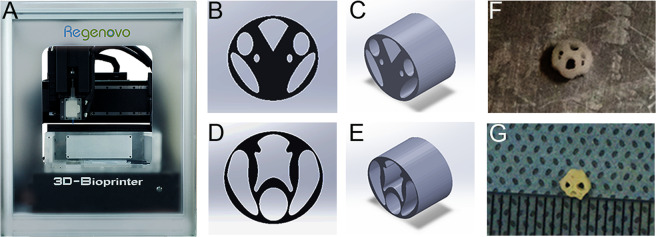
Design and manufacture of the 3D-C/SF scaffold. **A** A 3D bioprinter. Design of the original model (**B**) and its 3D presentation (**C**). **D**, **E** Final design model of the 3D-C/SF scaffold (**D**) and its 3D presentation (**F**). 3D-C/SF scaffold at −20 °C. **G** 3D-C/SF scaffold after freeze-drying

3D-C/SF scaffolds were fabricated by a 3D bioprinter (Regenovo Biotechnology Co., Ltd., Hangzhou, China) (Fig. 1A). Before printing in the present study, we designed a model with SolidWorks 2014 software (Dassault Systèmes). According to the anatomic structure of the spinal cord, the conduction bundle was used for the unit model design of the spinal cord scaffold. The scaffold model was an oval structure measuring 3.0 mm in transverse diameter, 2.8 mm in longitudinal diameter, and 3.00 mm in thickness. The spinal cord scaffold cross-section model and its 3D presentation were showed in Fig. 1B,C. After the third modification, the final model used to produce the scaffolds was showed in Fig. 1D, E. The molded 3D-C/SF scaffold was freeze-dried with four irregular channels (Fig. 1F, G). The hollow internal cylinders act as guidance channels for axonal growth [32–34].

### Characterization of scaffold

**Fig. 2:**
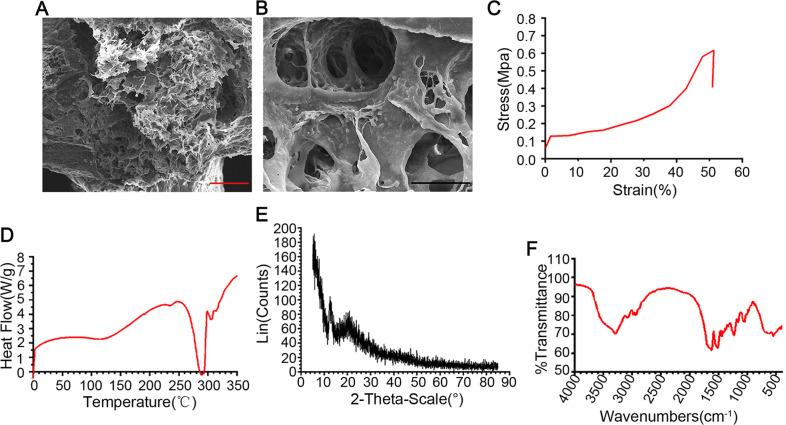
Scaffold characterization. **A**, **B** Field emission scanning electron microscopy images. **A** Magnification, ×400; scale bar, 25 µm. **B** Magnification, ×1500; scale bar, 10 μm. **C** Mechanical assays after tensile loading of the 3D-C/SF scaffold conformation. **D** Differential scanning calorimetry spectra of 3D-C/SF scaffold conformation, showing the amorphous nature of the 3D-C/SF scaffold. **E** X-ray diffraction. **F** FTIR spectra

The cross section of the 3D-C/SF scaffold resembles a honeycomb. The microporous nature of 3D-C/SF scaffold allows for communication between the holes (Fig. 2A, B). The Young’s modulus of the 3D-C/SF scaffold was 0.60 ± 0.12 MPa (Fig. 2C). The amorphous nature of the 3D-C/SF scaffold was characterized by DSC (Fig. 2D) and X-ray diffractometry (Fig. 2E). As shown in Fig. 2D, the 3D-C/SF scaffold exhibited three endothermic peaks with Joule heating after standardization: 1.75, 97.54, and 4.11 J/g at 228, 269, and 300 °C, respectively. As shown in the FTIR spectra (Fig. 2F), the O–H bond of the hydroxyl and the N–H bond of the amino possibly appeared at 3287 cm^−1^. A peak of 2800–3000 cm^−1^ may indicate C–H stretching vibrations of methyl or methylene; an absorption peak of 1600–1850 cm^−1^ may indicate C=O stretching vibration, C=C double bonds, the water peak, or the amino peak; and a strong absorption peak of 1000–1300 cm^−1^ can prove the existence of the C–O.

### In vivo degradation of 3D-C/SF scaffold

**Fig. 3:**
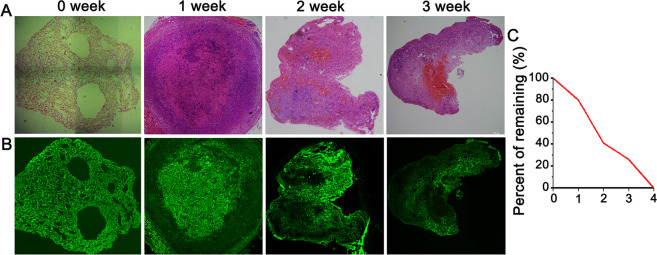
In vivo degradation. Degradation of 3D-C/SF scaffold was shown by HE (**A**) and spontaneous fluorescence (**B**). **C** The amount of 3D-C/SF scaffold remaining was quantified

Degradation of 3D-C/SF scaffold was shown by HE and spontaneous fluorescence. Figure 3A,B were HE and autofluorescence before implantation. Spontaneous fluorescence of 3D-C/SF is in green color. The green area reduced gradually indicating that scaffolds degraded in vivo. At 1 week after implantation, the amount of 3D-C/SF scaffold remaining was ~80%. At 2 weeks after implantation, the amount of 3D-C/SF scaffold remaining was 41%. At 3 weeks, the amount of 3D-C/SF scaffold remaining was only 26%. At 4 weeks post implantation, the 3D-C/SF scaffold was completely degraded and replaced by surrounding tissue (Fig. 3C).

### Improvement of locomotor function

**Fig. 4:**
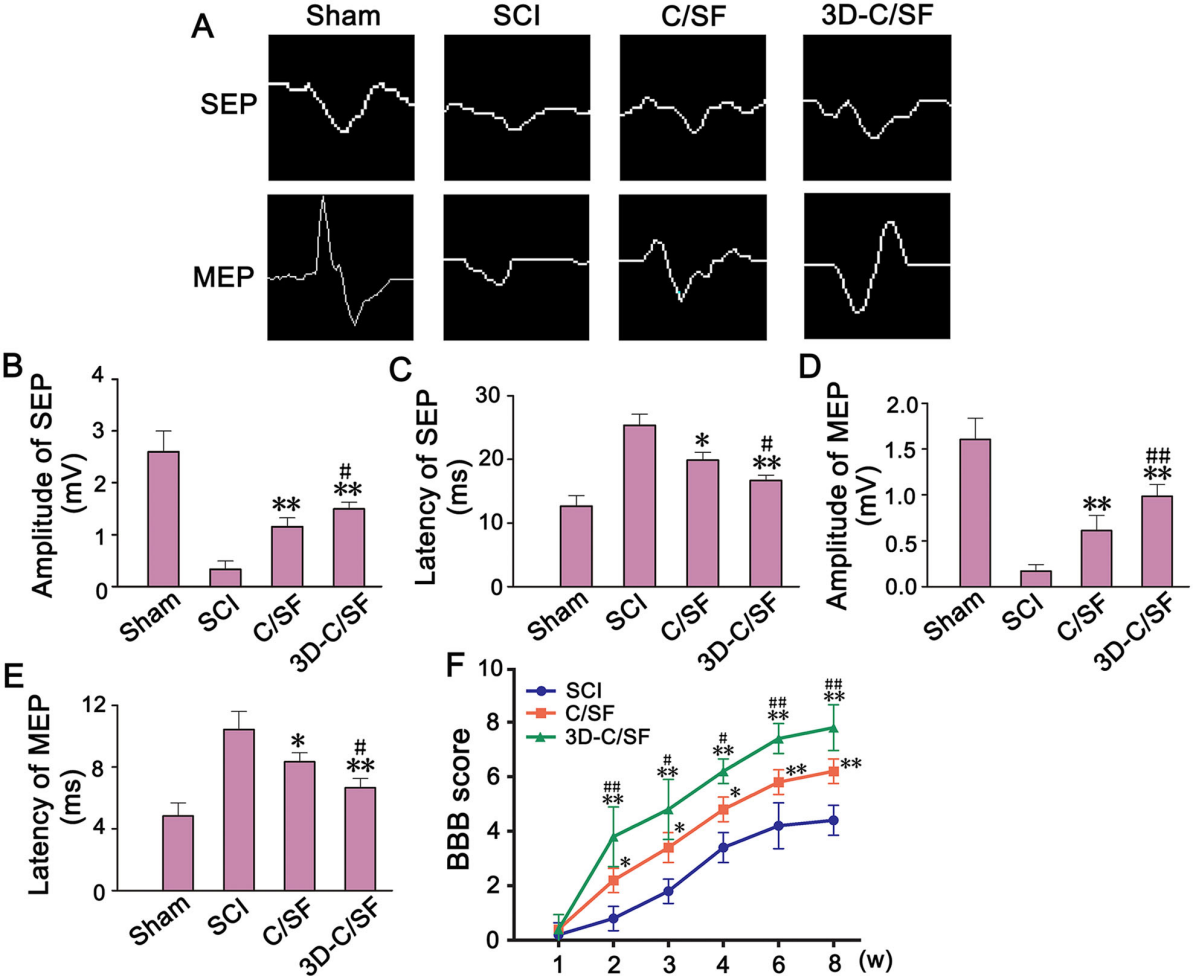
Electrophysiologic and behavior outcomes. **A**–**E** Motor evoked potentials (MEP) were obtained by electrophysiological analysis. Representative image of MEP and SEP (**A**). The latency (**B**) and amplitude (**C**) of MEP. The latency (**D**) and amplitude (**E**) of SEP. **F** The BBB score was obtained during the course of recovery after SCI. *P < 0.05, **P < 0.01 vs SCI group. ^#^*P* < 0.05, ^##^*P* < 0.01 vs SCI + C/SF group

As widely used clinical evaluation parameters, the amplitude and latency of the MEP and SEP are conventionally presumed to reflect the number of excited axons and the conduction velocity of the nerve, respectively. At 8 weeks after injury, 3D-C/SF implant showed higher amplitude and shorter latency compared with SCI and C/SF group both in SEP (Fig. 4A–C) and MEP (Fig. 4 A, D, E). The BBB score was 0 when the spinal cord was completely transected. Two weeks after transplantation, the locomotor performance gradually improved in each group. At 8 weeks, the average BBB score in the 3D-C/SF group was 7.91 ± 0.68, which was significantly higher than that in SCI and C/SF group (Fig. 4F). These results suggested that the 3D-C/SF scaffold contributed to improvement in the locomotor function after SCI.

### Magnetic resonance imaging (MRI)

**Fig. 5:**
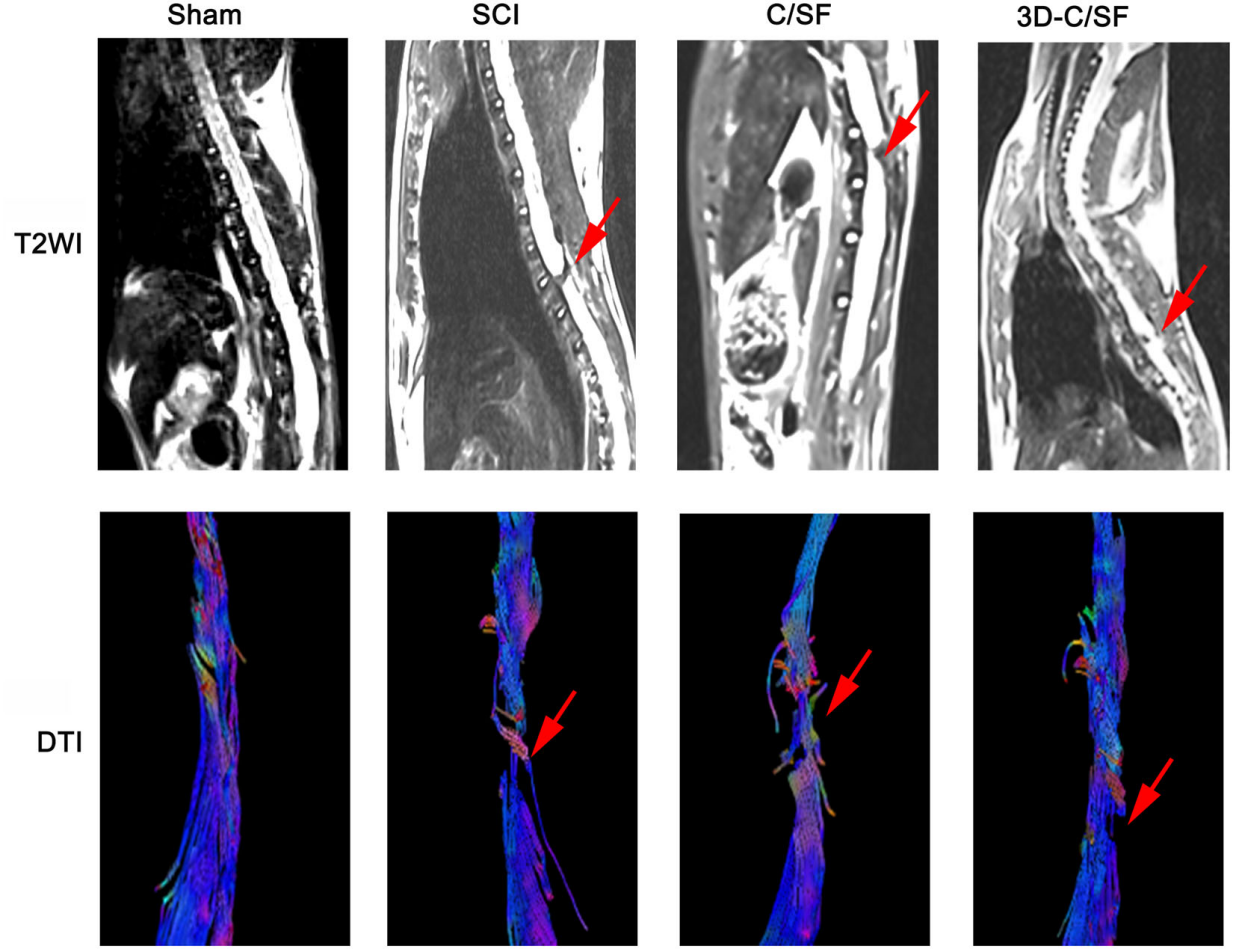
MRI imaging. T2WI and DTI images of spinal cord in each group at 8 weeks after implantation. All red arrows showed the damage area at the T10 spinal cord level

At the end of the 8 week, recovery of the spinal cord defect site was observed by MRI. As shown by the T2 weighted image (T2W1) and DTI images, the axonal regeneration was significantly greater in the 3D-C/SF group than SCI and C/SF group. The proximal and distal sites were partially reconnected by regenerated axons in 3D-C/SF group. DTI allows for visualization of the spinal cord regeneration and is a good technique with which to confirm SCI recovery (Fig. 5).

### Axonal regeneration

**Fig. 6:**
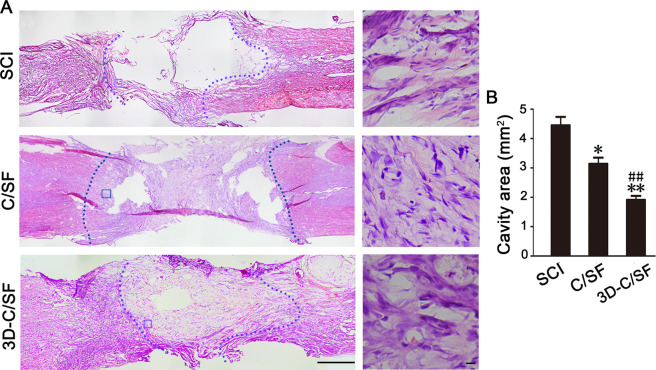
HE staining at 8 weeks post injury. **A** Low magnification of spinal cord sections (left) and higher magnification of the white boxed sections (right). **B** Higher magnification of the boxed sections. Scale bar, 50 µm

**Fig. 7:**
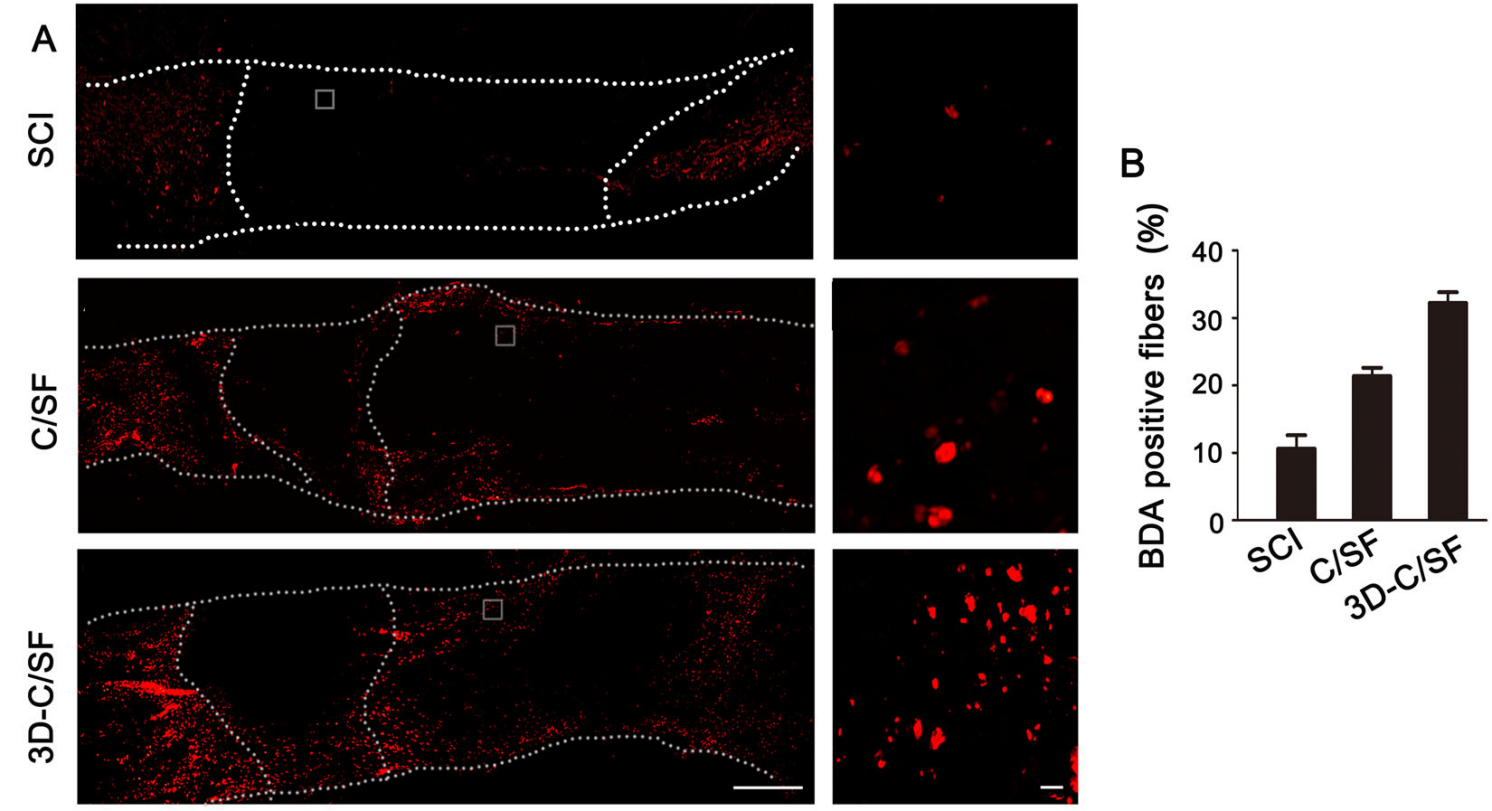
Corticospinal tract tracing at 8 weeks after injury. **A** BDA-positive fibers in rostral spinal cord sections. The right panel was the higher magnification of the box in left panel. Panel **B** is the quantification of BDA-positive fibers of **A** within each group. Scale bar, 50 µm

**Fig. 8:**
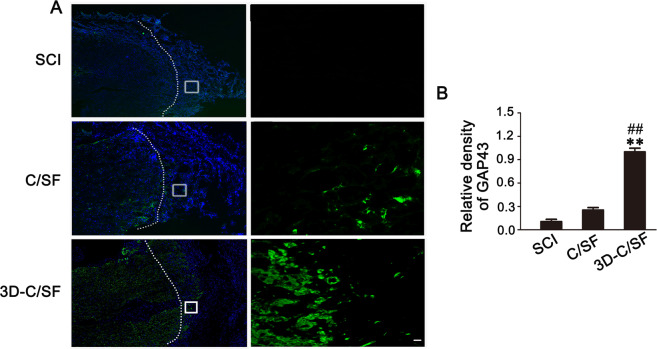
**A** GAP43-positive profiles in the horizontal spinal cord sections at low magnification (left) and high magnification (right) in the three groups. **B** The quantification of the relative density of GAP43. The dashed lines indicate rostral interfaces. Scale bar, 50 µm

The improvement in functional recovery in response to the 3D-C/SF scaffold may have been a result of anatomical and pathophysiological changes. Eight weeks post injury, HE analysis was performed on longitudinal sections of the injured spinal cords. We observed lesions and disordered structures in the SCI and C/SF group, while fewer lesions and disordered structures were present in the 3D-C/SF group (Fig. 6A, B). We found that the 3D-C/SF group exhibited more BDA caudal to the injury site than the SCI and C/SF group (Fig. 7A, B). We also observed that the 3D-C/SF group showed more GAP43-positive profiles at the lesion site than the SCI and C/SF group (Fig. 8A, B). These results indicated that the 3D-C/SF treatments improved axonal regeneration, a critical component of functional recovery in SCI.

### Nerve fiber regeneration and myelination

**Fig. 9:**
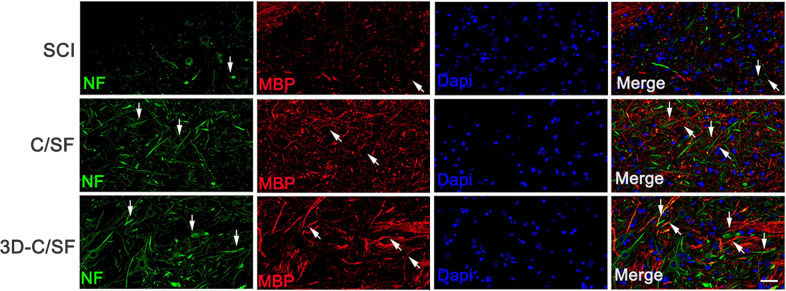
Representative images of NF (green) and MBP (red) double immunofluorescence staining at the injury site. The white arrows indicated NF-positive nerve fibers or MBP-positive myelin-like structures. Scale bar, 50 µm

At 8 weeks after SCI, 3D-C/SF group demonstrated more NF-positive nerve fibers in the injury site compared to SCI and C/SF group (Fig. 9). Furthermore, we found that the number of NF-positive nerve fibers wrapped by MBP-positive myelin sheath structures at the injury site was increased in 3D-C/SF group compared to SCI and C/SF group (Fig. 9). These results suggested that the 3D-C/SF treatments promote nerve fiber regeneration and myelination after SCI.

## Discussion

In the present study, we achieved a rapid prototyping at low temperatures and time-controllable degradation in vivo by a favorable C/SF biodegradable material ratio and improved scaffold properties. The C/SF implants based on 3D printing accurately simulated the spatial structure of the spinal tracts, traversed glial scar tissue, facilitated nerve fiber regeneration and myelination, and improve locomotor function.

Recovery of neurological function after SCI is still a problem for clinicians and scientists. Difficulty in establishing axonal regeneration is the main factor that prevents recovery of neurological function [35]. The suppressive (physical and chemical) microenvironment at the injury site blocks axonal regeneration [36]. Tissue engineering techniques have recently been proposed to solve this problem [8]. Excellent biocompatible materials and well-designed 3D spinal microcatheters contribute to bridging of axons through the scar barrier, which play important roles in improving the suppressive microenvironment and promoting axonal regeneration [37].

Recently, common models in tissue engineering for the study of neuronal regeneration after SCI in rats include transection, segment resection, hemisection, and contusion. However, each model can only simulate the complex pathophysiology of a certain type of SCI. Any model can not completely reflect the changes in human SCI anatomy and nerve function [38, 39]. There is a certain disconnect between what happens clinically with SCI patients and what is done in the laboratory and how the two are translated. For example, although the transection model is a good, clean model for observing scaffolds, complete SCI injury in the clinical population with spinal cord transection is relatively rare. In essence, most clinical spinal cord injuries are contusive.

The following conditions should be met for the study of SCI animal models: firstly, the model needs to be stable. Within a period of time after SCI without any treatment, there should be no significant differences in locomotor function assessment or any indicators for assessing repair. Secondly, the transection of the nerve fiber bundle is complete, or the residual fibers can be quantified before performing any treatment. Differences can cause controversy in experimental results. The repeatability and availability of animal models should also be considered [40]. Considering the scaffold implantation in this study and meeting the above conditions, the transection model may be an appropriate model for the study of biomaterial scaffolds for SCI repair. In some studies, aneurysm clips were used to establish SCI model, and hydrogel was injected into the injury site after SCI [41]. The location choice of the injury between T9 and T10 should also be motivated. The type of surgery may influence the repair/regeneration of SCI. There are two types of surgery: the scaffold should be implanted immediately after laminectomy and spinal myelectomy. Another surgical method is to wait a period of time between injury and repair/regenerative surgery to mimic the most common clinical cases. After a certain period of time after SCI, glial scars will be produced which hinder the repair of SCI. Therefore, the repair effect of implanting the scaffold immediately after injury is better than that of waiting a period of time after the injury and then implanting the scaffold. Implanting scaffolds immediately after laminectomy and spinal myelectomy was the more preferred choice for the studies of scaffolds for SCI repair [42, 43].

Conduits such as those described in this study accurately guide nerve tracts, such as those in the corticospinal tract, to traverse the injury site and arrive at the corresponding distal corticospinal tract [44]. Structural analysis has shown that the spinal cord comprises nerve fiber tracts with a relatively fixed multistrand structure, ordered arrangement, and specific function. Nerve tracts are mainly distributed within the white matter near the outer edge of the spinal cord, and these tracts have different cross-sectional areas and irregular shapes. Injury to these nerve fiber tracts is a direct cause of physical impairment after SCI [45]. Following SCI, the axons of the nerve fiber tracts regenerate and become prolonged, traverse the injury site, and connect to the axons at the distal injury site; this process can restore the conduction of electroneurographic signals.

Studies have shown that three-dimensional bioprinting C/SF scaffold combined with neural stem cells promoted nerve regeneration after SCI, which mainly proved that neural stem cells were beneficial to the repair of SCI [46]. Our study complemented the shortcomings of previous studies: when preparing a new structural scaffold, it is necessary to first compare it with conventional structural scaffolds. Our study demonstrated that compared with the C/SF scaffold prepared by traditional freeze-drying technology, the 3D printing C/SF scaffold had better mechanical properties and was more conducive to nerve fiber regeneration and myelination after SCI to improve locomotor function. Our study and previous studies respectively make up for each other’s shortcomings and jointly show that 3D printing C/SF scaffolds were beneficial to the repair of SCI and had good biocompatibility, and could be combined with neural stem cells to ameliorated axon regeneration and neurological recovery after SCI.

Conventionally prepared and uniformly distributed porous conduits cannot provide such precise guidance for the nerve tracts [47]. The spinal microcatheter designed in the present study can be divided into the fasciculus gracilis, fasciculus cuneatus, lateral corticospinal tract, and lateral spinothalamic tract (Fig. 1A, B). The 3D optimized structure combines the lateral corticospinal tract and lateral spinothalamic tract (Fig. 1C, D). This design simulates the physical structure of the rat spinal cord nerve tracts and is fit for spatial connection of white matter tracts. In this study, we were able to control the complex shape of the catheter and fine internal structure using 3D printing technology, optimize the catheter structure by finite element analysis, and achieve fine quality control of the catheter.

The characteristics of the scaffold are directly related to the effect of nerve repair. An ideal scaffold should have good biocompatibility, good neural induction, and suitable mechanical properties [48]. Collagen is a constitutive protein with the highest content in the extracellular matrix, and is involved in many cellular functions. Collagen has good cell compatibility. Dai [49, 50] considered that a nerve growth factor-activated collagen scaffold could promote the recovery of neurological function in the spinal cords of rats and Beagle dogs. However, collagen also has some disadvantages, such as a poor ability to assume a 3D multilayer porous structure, instability, poor biomechanics, and rapid in vivo degradation. SF has good biocompatibility, high mechanical strength, a stable structure, and a slow degradation rate. Thus, collagen and silk fibroin have complementary advantages [51]. Natural biological materials mainly include natural fibers, biological tissues, structural proteins, biological minerals, and other materials [8]. Natural materials include hyaluronic acid, collagen, fibrin, alginate, chitosan, polyhydroxybutyrate, and methyl cellulose hydrogel, which can be used as scaffolds alone or as synthetic scaffolds made of natural materials. Therefore, these natural materials and their synthetic natural biological scaffolds have good biocompatibility, cell affinity and degradability, and can better promote cell adhesion and proliferation [8, 52]. Hyaluronic acid widely exists in the extracellular interstitial space, which can greatly reduce the migration and differentiation of lymphocytes at the injured site, prevent its inflammatory tendency, prevent the phagocytosis of inflammatory cells, and thus reduce the secondary injury of SCI and promote the regeneration of spinal cord nerve tissue after injury [53, 54]. However, scaffolds made of hyaluronic acid have poor adhesion to seed cells, which has been improved in recent studies. Some scholars added Laminin (LA) to scaffolds made of hyaluronic acid, or coated with lysine coating [55], which greatly improved adhesion ability. However, due to the weak support strength of hyaluronic acid scaffold and its high degradation rate were not improved, it was less used in the repair of SCI. Other natural materials were used to make scaffolds to repair SCI include alginate, chitosan, and hydrogel [56, 57]. These studies have shown that compared to the scaffolds made of artificial materials, the scaffolds made of natural materials have improve the adhesion ability of seed cells, have good biocompatibility and degradation and non-toxicit, rolong the survival time of seed cells in vivo, increase the rate of seed cells into nerve cells differentiation, can better promote tissue regeneration and functional rehabilitation [58]. Some scholars have found that the original materials have some shortcomings when they are directly used for repair: the mechanical support is weak, the regenerated nerve tissue cannot be guided through the injured area effectively, and the degradation rate is not synchronized with the body’s rehabilitation [57, 58]. If the scaffold is reshaped with natural materials, the composite scaffold will also face difficulties in hardness and degradation to match the progress of tissue repair, and the porosity is difficult to fully match the regrowth of axons. Faced with these problems, scaffolds made of natural materials need to be improved to repair SCI.

In the present study, we used a C/SF composite, altered the proportion of the mixed solution, and created a biomaterial that can be formed at −20 °C rapidly and uniformly. A microcatheter stent with a precision of 50 µm can be printed with a 3D printer. Due to the nature of the material, Young’s modulus at 0.60 ± 0.12 MPa is more suitable for the growth of nerve cells [59]. DSC and X-ray diffractometry data revealed that this material has three different melting peaks. X-ray films showed no obvious crystallization, high uniformity of the material, and high stability. FTIR spectra data showed that the composite has appropriate fat-soluble and water-soluble chemical bonds and is suitable for the adhesion and growth of nerve cells.

The standard SCI model was the premise of this study. We used a rat model of SCI involving 3-mm complete transection at the T10 level [4]. DTI images of postoperative MRI, SEP, and MEP data and BBB scores confirmed that this SCI model was successful. Eight weeks after surgery, DTI images in the 3D-C/SF group revealed that partial nerve tracts were connected to the distal spinal cord by the injury site. The CMEPs in the 3D-C/SF group had a higher amplitude and shorter latency than those in the control group. Two weeks after surgery, the BBB scores showed a gradual recovery trend in both groups. The recovery was noticeably better in the 3D-C/SF group than in the control group, indicating that the 3D-C/SF scaffolds contributed to the reconstruction of motor function after SCI.

The pathophysiological changes provided more proof of functional recovery. At 8 weeks, a smaller area of damage and a relatively orderly structure indicated that the material was conducive to reconstruction of the damaged tissue and establishment of an orderly arrangement of the neural network in the 3D-C/SF group. GFAP staining showed that 3D-C/SF controlled the local suppressive microenvironment relatively well and reduced the formation of glial scarring. Moreover, the BDA results further verified that the nerve tracts partially traversed the glial scar and became linked to the distal axons. More interestingly, the 3D-C/SF scaffolds contributed to the reconstruction and regeneration of synapses, which is anatomically important for the recovery of neurological function.

Microcatheter scaffolds play an important role in nerve repair after SCI. In particular, the fine structure of these microcatheters can guide the docking and remodeling of injured axon termina [44]. The characteristics of the microcatheter material also determine the local microenvironment; time control of biological degradation is a particularly important factor in the selection of catheter materials [58]. The structure and function can be effectively perfectly repaired, only when axonal regeneration can be guided in a certain time, the material is gradually degraded in a certain period so that the material cannot hinder further axonal remodeling and formation of the neural network.

Our study has some limitations that we did not study the use of scaffolds combined with nerve cells and nerve growth factors for SCI repair. These are necessary to regulate the local microenvironment and promote nerve regeneration. These factors will be investigated in our future studies.

## Conclusions

Following completely transected SCI, 3D-C/SF implants based on the structural design of nerve tracts help to guide the reorientation of the spinal cord tracts and promote axonal connection. The 3D-C/SF implants achieve a good repair effect within an appropriate time and contribute to nerve regeneration and stable recovery of motor function. Nevertheless, further modification of the 3D-C/SF performance and 3D space structure is necessary to obtain even better clinical effects.

## References

[1] Singh A, Tetreault L, Kalsiryan S, Nouri A, Fehlings MG (2014). Global prevalence and incidence of traumatic spinal cord injury. Clin Epidemiol.

[2] Silva NA, Sousa N, Reis RL, Salgado AJ (2014). From basics to clinical: a comprehensive review on spinal cord injury. Prog Neurobiol.

[3] Li C, Zhang X, Cao R (2012). Allografts of the acellular sciatic nerve and brain-derived neurotrophic factor repair spinal cord injury in adult rats. PLoS ONE.

[4] Lu P, Wang Y, Graham L (2012). Long-distance growth and connectivity of neural stem cells after severe spinal cord injury. Cell.

[5] Sharp KG, Dickson AR, Marchenko SA (2012). Salmon fibrin treatment of spinal cord injury promotes functional recovery and density of serotonergic innervation. Exp Neurol.

[6] Krishna V, Konakondla S, Nicholas J, Varma A, Kindy M, Wen X (2013). Biomaterial-based interventions for neuronal regeneration and functional recovery in rodent model of spinal cord injury: a systematic review. J Spinal Cord Med.

[7] Friedman JA, Windebank AJ, Moore MJ, Spinner RJ, Currier BL, Yaszemski MJ (2002). Biodegradable polymer grafts for surgical repair of the injured spinal cord. Neurosurgery..

[8] Madigan NN, Mcmahon S, O’Brien T, Yaszemski MJ, Windebank AJ (2009). Current tissue engineering and novel therapeutic approaches to axonal regeneration following spinal cord injury using polymer scaffolds. Respir Physiol Neurobiol.

[9] Álvarez Z, Castaño O, Castells AA (2014). Neurogenesis and vascularization of the damaged brain using a lactate-releasing biomimetic scaffold. Biomaterials..

[10] Chen BK, Knight AM, Madigan NN (2011). Comparison of polymer scaffolds in rat spinal cord: a step toward quantitative assessment of combinatorial approaches to spinal cord repair. Biomaterials..

[11] Teng YD, Lavik EB, Qu X (2002). Functional recovery following traumatic spinal cord injury mediated by a unique polymer scaffold seeded with neural stem cells. Proc Natl Acad Sci USA.

[12] Walker PA, Aroom KR, Jimenez F (2009). Advances in progenitor cell therapy using scaffolding constructs for central nervous system injury. Stem Cell Rev Rep.

[13] Wong DY, Krebsbach PH, Hollister SJ (2008). Brain cortex regeneration affected by scaffold architectures. J Neurosurg.

[14] Michalski MH, Ross JS (2014). The shape of things to come: 3D printing in medicine. JAMA.

[15] Schubert C, van Langeveld MC, Donoso LA (2014). Innovations in 3D printing: a 3D overview from optics to organs. Br J Ophthalmol.

[16] Liu S, Said G, Tadie M (2001). Regrowth of the rostral spinal axons into the caudal ventral roots through a collagen tube implanted into hemisected adult rat spinal cord. Neurosurgery..

[17] Chen JL, Yin Z, Shen WL (2010). Efficacy of hESC-MSCs in knitted silk-collagen scaffold for tendon tissue engineering and their roles. Biomaterials..

[18] Shen W, Chen X, Hu Y (2014). Long-term effects of knitted silk-collagen sponge scaffold on anterior cruciate ligament reconstruction and osteoarthritis prevention. Biomaterials..

[19] Sun K, Li H, Li R, Nian Z, Li D, Xu C (2015). Silk fibroin/collagen and silk fibroin/chitosan blended three-dimensional scaffolds for tissue engineering. Eur J Orthop Surg Traumatol.

[20] Chen X, Qi YY, Wang LL (2008). Ligament regeneration using a knitted silk scaffold combined with collagen matrix. Biomaterials..

[21] Levin B, Redmond SL, Rajkhowa R, Eikelboom RH, Atlas MD, Marano RJ (2013). Utilising silk fibroin membranes as scaffolds for the growth of tympanic membrane keratinocytes, and application to myringoplasty surgery. J Laryngol Otol.

[22] Madduri S, Papaloïzos M, Gander B (2010). Trophically and topographically functionalized silk fibroin nerve conduits for guided peripheral nerve regeneration. Biomaterials..

[23] Huang W, Begum R, Barber T (2012). Regenerative potential of silk conduits in repair of peripheral nerve injury in adult rats. Biomaterials..

[24] Tang X, Xue C, Wang Y, Ding F, Yang Y, Gu X (2012). Bridging peripheral nerve defects with a tissue engineered nerve graft composed of an in vitro cultured nerve equivalent and a silk fibroin-based scaffold. Biomaterials..

[25] Yuhui R, Hong L, Jinrong Y (2005). Preparation of 3D fibroin/chitosan blend porous scaffold for tissue engineering via a simplified method. Macromol Biosci.

[26] Xu Y, Zhang Z, Chen X, Li R, Li D, Feng S (2016). A silk fibroin/collagen nerve scaffold seeded with a co-culture of Schwann cells and adipose-derived stem cells for sciatic nerve regeneration. PLoS ONE.

[27] Wang N, Xiao Z, Zhao Y, Wang B, Li X, Li J (2018). Collagen scaffold combined with human umbilical cord-derived mesenchymal stem cells promote functional recovery after scar resection in rats with chronic spinal cord injury. J Tissue Eng Regen Med.

[28] Shreiber DI, Barocas VH, Tranquillo RT (2003). Temporal variations in cell migration and traction during fibroblast-mediated gel compaction. Biophys J.

[29] Shinozaki M, Yasuda A, Nori S (2013). Novel method for analyzing locomotor ability after spinal cord injury in rats: technical note. Neurol Med Chir.

[30] Gelderd JB, Chopin SF (2010). The vertebral level of origin of spinal nerves in the rat. Anat Rec-Adv Integr Anat Evolut Biol.

[31] Basso DM, Beattie MS, Bresnahan JC (1995). A sensitive and reliable locomotor rating scale for open field testing in rats. J Neurotrauma.

[32] Koffler J, Zhu W, Qu X, Platoshyn O, Dulin JN, Brock J (2019). Biomimetic 3D-printed scaffolds for spinal cord injury repair. Nat Med.

[33] Joung D, Truong V, Neitzke CC, Guo SZ, Walsh PJ, Monat JR, et al. 3D printed stem-cell derived neural progenitors generate spinal cord scaffolds. Adv Funct Mater. 2018;28. 10.1002/adfm.201801850 PMC7319181 32595422

[34] Kaplan B, Merdler U, Szklanny AA, Redenski I, Guo S, Bar-Mucha Z (2020). Rapid prototyping fabrication of soft and oriented polyester scaffolds for axonal guidance. Biomaterials..

[35] Fagoe ND, Heest JV, Verhaagen J (2014). Spinal cord injury and the neuron-intrinsic regeneration-associated gene program. Neuromolecular Med.

[36] Calmels P, Mick G, Perrouin-Verbe B, Ventura M (2009). Neuropathic pain in spinal cord injury: identification, classification, evaluation. Ann Phys Rehabilitation Med.

[37] Kim M, Park SR, Choi BH (2014). Biomaterial scaffolds used for the regeneration of spinal cord injury (SCI). Histology &. Histopathology..

[38] Sedý J, Urdzíková L, Jendelová P, Syková E (2008). Methods for behavioral testing of spinal cord injured rats. Neurosci Biobehav Rev.

[39] Li XF, Dai LY (2009). Three-dimensional finite element model of the cervical spinal cord: preliminary results of injury mechanism analysis. Spine.

[40] Wang F, Huang SL, He XJ, Li XH (2014). Determination of the ideal rat model for spinal cord injury by diffusion tensor imaging. Neuroreport.

[41] He Z, Zang H, Zhu L, Huang K, Yi T, Zhang S (2019). An anti-inflammatory peptide and brain-derived neurotrophic factor-modified hyaluronan-methylcellulose hydrogel promotes nerve regeneration in rats with spinal cord injury. Int J Nanomed.

[42] Lai BQ, Feng B, Che MT, Wang LJ, Cai S, Huang MY (2018). A modular assembly of spinal cord-like tissue allows targeted tissue repair in the transected spinal cord. Adv Sci.

[43] Li X, Zhao Y, Cheng S, Han S, Shu M, Chen B (2017). Cetuximab modified collagen scaffold directs neurogenesis of injury-activated endogenous neural stem cells for acute spinal cord injury repair. Biomaterials..

[44] Hellal F, Hurtado A, Ruschel J (2011). Microtubule stabilization reduces scarring and enables axon regeneration after spinal cord injury. Science..

[45] Baker SN, Zaaimi B, Fisher KM, Edgley SA, Soteropoulos DS (2015). Pathways mediating functional recovery. Prog Brain Res.

[46] Jiang JP, Liu XY, Zhao F, Zhu X, Li XY, Niu XG (2020). Three-dimensional bioprinting collagen/silk fibroin scaffold combined with neural stem cells promotes nerve regeneration after spinal cord injury. Neural Regen Res.

[47] Gros T, Sakamoto JS, Blesch A, Havton LA, Tuszynski MH (2010). Regeneration of long-tract axons through sites of spinal cord injury using templated agarose scaffolds. Biomaterials..

[48] Kaneko A, Matsushita A, Sankai Y (2015). A 3D nanofibrous hydrogel and collagen sponge scaffold promotes locomotor functional recovery, spinal repair, and neuronal regeneration after complete transection of the spinal cord in adult rats. Biomed Mater.

[49] Han Q, Jin W, Xiao Z (2010). The promotion of neural regeneration in an extreme rat spinal cord injury model using a collagen scaffold containing a collagen binding neuroprotective protein and an EGFR neutralizing antibody. Biomaterials..

[50] Han S, Wang B, Jin W (2015). The linear-ordered collagen scaffold-BDNF complex significantly promotes functional recovery after completely transected spinal cord injury in canine. Biomaterials..

[51] Kundu B, Rajkhowa R, Kundu SC, Wang X (2013). Silk fibroin biomaterials for tissue regenerations. Adv Drug Deliv Rev.

[52] Potter W, Kalil RE, Kao WJ (2008). Biomimetic material systems for neural progenitor cell-based therapy. Front Biosci.

[53] Burd DA, Greco RM, Regauer S, Longaker MT, Siebert JW, Garg HG (1991). Hyaluronan and wound healing: a new perspective. Br J Plast Surg.

[54] Ozgenel GY, Filiz G (2003). Effects of human amniotic fluid on peripheral nerve scarring and regeneration in rats. J Neurosurg.

[55] Tian WM, Hou SP, Ma J, Zhang CL, Xu QY, Lee IS (2005). Hyaluronic acid-poly-D-lysine-based three-dimensional hydrogel for traumatic brain injury. Tissue Eng.

[56] Nisbet DR, Crompton KE, Horne MK, Finkelstein DI, Forsythe JS (2010). Neural tissue engineering of the CNS using hydrogels. J Biomed Mater Res Part B Appl Biomater.

[57] Samadikuchaksaraei A (2007). An overview of tissue engineering approaches for management of spinal cord injuries. J Neuroeng Rehabil.

[58] Sakiyama-Elbert S, Johnson PJ, Hodgetts SI, Plant GW, Harvey AR (2012). Scaffolds to promote spinal cord regeneration. Handb Clin Neurol.

[59] Discher D (2006). Matrix elasticity directs stem cell lineage specification. Cell..

